# Cervical extension of pancreatic pseudocyst: An unusual cause of neck stiffness and dysphagia

**DOI:** 10.4102/sajr.v26i1.2385

**Published:** 2022-05-09

**Authors:** Sneha Harish C, Rashmi Dixit, Sapna Singh, Anjali Prakash

**Affiliations:** 1 Department of Radiodiagnosis, Maulana Azad Medical College and Associated Lok Nayak Hospital, New Delhi, India

**Keywords:** pancreatitis, pseudocyst, cervical extension, dysphagia, neck stiffness, mediastinal pseudocyst, computed tomography

## Abstract

Pancreatic pseudocyst is a common complication that can occur following acute or chronic pancreatitis. Commonly, they are peripancreatic in location. Rarely, they can extend to the mediastinum, and further extension to the neck is even rarer. A 55-year-old man who presented with neck stiffness and dysphagia and on imaging, was found to have a cystic lesion in the neck. Aspiration of the lesion revealed raised amylase levels suggestive of a pancreatic pseudocyst.

## Introduction

Pancreatic pseudocyst is a very common complication that can occur following either acute or chronic pancreatitis. It is seen in about 30% – 40% of patients with chronic pancreatitis.^1^ Commonly, these cysts are found in peripancreatic locations. Rarely, they can extend into the mediastinum through anatomical defects in the diaphragm.^2,3,4,5,6^ Further extension to the neck is even rarer and has been described in a few case reports only.^7,8,9^ These can present with neck swelling or mass effect on the adjacent structures, and patients may present with complaints of dysphagia, chest pain or shortness of breath. Due to the varied clinical presentation, diagnosis is often challenging. Cross-sectional imaging such as CT is an excellent tool to establish the diagnosis.

This report describes the case of a 55-year-old man with cervical extension of a pancreatic pseudocyst.

## Case report

A 55-year-old male patient presented to the Emergency Department with complaints of neck stiffness and dysphagia for one week. There was no history of trauma or fever. Occasional alcohol intake was documented. At examination, the patient had limited neck movements along with tachypnoea. However, the lung auscultation findings were normal. The abdomen was soft and non-tender with no obvious palpable lump.

**FIGURE 1:**
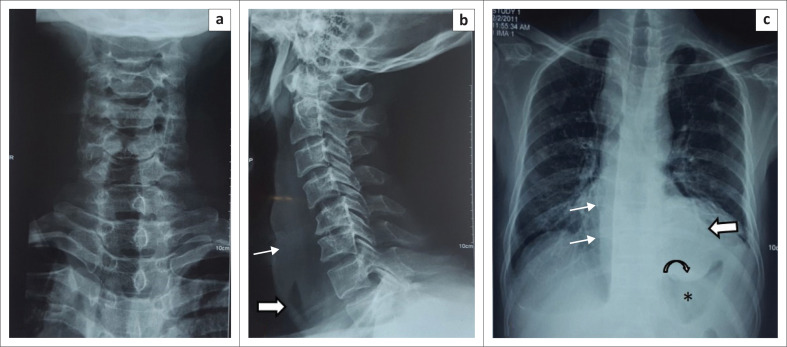
Radiograph of the neck, anteroposterior (a) and lateral (b), demonstrates anterior displacement of the airway and oesophagus (thick arrow) and widening of the prevertebral soft tissue (thin arrow). The frontal chest radiograph (c) indicates displacement of the right paraspinal stripe (thin arrows) and a retrocardiac opacity (thick arrow) indenting (curved arrow) the gastric fundus (asterisk).

A radiograph of the neck and cervical spine was obtained (Figure 1a and b), which indicated thickened prevertebral soft tissue causing anterior displacement of the airway. Degenerative changes were seen in the cervical spine. No evidence of discitis or vertebral osteomyelitis was seen. A chest radiograph acquired at the same time (Figure 1c) revealed a homogenous opacity causing displacement of the right paratracheal stripe and thickening of the paravertebral stripe. Another near-homogenous opacity was seen in the retrocardiac region, silhouetting the left hemidiaphragm. Given the radiographic findings and clinical scenario, the possibility of retropharyngeal abscess with mediastinal extension was considered.

**FIGURE 2:**
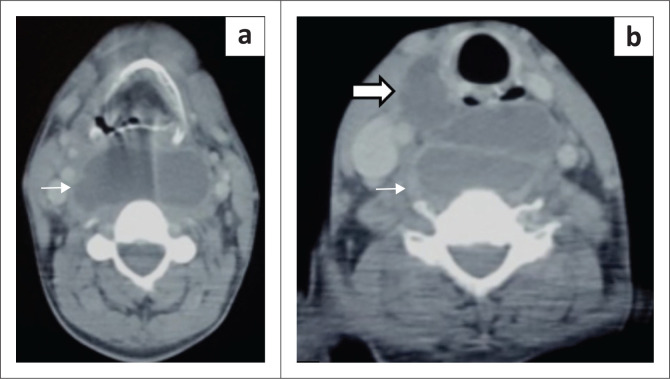
Axial contrast-enhanced CT scan of the neck, at the level of the hyoid bone (a) and at the level of sixth cervical vertebra (b), reveals a peripherally enhancing cystic lesion (thin arrows in a and b) involving the retropharyngeal and prevertebral space with extension to the anterior cervical and visceral space (thick arrow in b).

**FIGURE 3:**
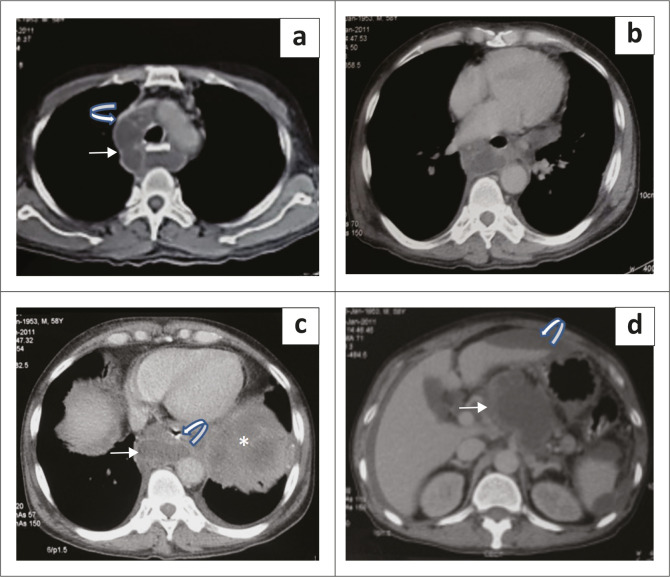
Axial sections of contrast-enhanced CT scan of the chest and abdomen (a–d) show the pseudocyst in the visceral compartment of the mediastinum abutting the superior vena cava (curved arrow in a) and descending thoracic aorta, with anterior displacement of the oesophagus (curved arrow in c). It is extending into the abdomen through the oesophageal hiatus (white arrow in c) where it is seen arising from the body of pancreas (white arrow in d). Ascites (curved arrow in d), bilateral pleural effusions and left lower lobe consolidation (asterisk in c) are also noted.

A contrast-enhanced CT scan of the neck and chest was conducted. It revealed a hypodense peripherally enhancing cystic lesion involving the prevertebral and retropharyngeal spaces of the neck, extending from the base of the skull to the level of the thoracic inlet (Figure 2a and b). Further caudally, it was seen to extend into the mediastinum, involving the visceral compartment, causing anterior displacement of the trachea and oesophagus (Figure 3a–c). More distally, the lesion was seen to extend through the oesophageal hiatus of the diaphragm into the abdomen, involving the lesser sac and body of pancreas (Figure 3d).

On subsequent probing, the patient provided a history of acute pain in the epigastric region eight weeks prior, which gradually subsided over a few days. Hence, the diagnosis of pancreatic pseudocyst extending to the mediastinum and neck was made.

As the patient was symptomatic, the cervical cyst was drained intraorally. Analysis of the aspirate indicated leucocytes and raised amylase levels (55 043 U/L), confirming the diagnosis. The pseudocyst resolved and the patient’s recovery was uneventful.

## Discussion

Pancreatic pseudocysts are common complications of both acute and chronic pancreatitis. The aetiology of pancreatitis and hence pseudocyst includes excessive alcohol consumption, biliary tract pathologies and trauma.^1^ In the paediatric population, additional causes include genetic abnormalities such as cystic fibrosis, pancreatic anomalies, ingestion of medications such as antiepileptics and metabolic disorders.^10^ Following an episode of acute pancreatitis, up to four weeks from the onset of pain, fluid collections associated with interstitial oedematous pancreatitis are defined as acute peripancreatic fluid collections according to the revised Atlanta classification for pancreatitis. If the fluid fails to resorb after four weeks and develops a mature wall, the term pseudocyst is used.^11^ Pathologically, a pseudocyst of the pancreas consists of a fluid collection surrounded by fibrous tissue with no epithelial lining.^12^ The fluid is rich in amylase, lipase and other enzymes produced by the pancreas. The symptoms are usually non-specific, but the possibility of pseudocyst formation should be considered in patients with persistent abdominal pain or a palpable mass in the abdomen following an episode of pancreatitis.^1^ Serum analysis is not reliable, as patients may or may not have elevated serum lipase and amylase levels and/or raised serum bilirubin levels.^1^

Pancreatic pseudocysts are commonly peripancreatic in location, involving the lesser sac. Occasionally, pancreatic pseudocysts can occur at atypical locations like the splenic hilum or within the spleen and mediastinum. Extension further cranially to the neck resulting in retropharyngeal collections is fairly uncommon and has only been described in a few reports.^6,8,9,13^ One case report also describes a pseudocyst presenting as an inguinoscrotal swelling.^14^ These atypical locations cause a diagnostic dilemma due to the varied clinical presentations. Hence a thorough clinical history along with cross-sectional imaging is required to make the diagnosis.

Mediastinal extension of a pancreatic pseudocyst although previously reported, is a relatively rare entity.^2,3,4,5,6^ A case of an isolated mediastinal pseudocyst with no obvious communication with the pancreas has also been reported.^15^ The most common location for a mediastinal pseudocyst is the posterior mediastinum, although they can be seen in the anterior or middle mediastinum. It occurs when there is extension of the fluid from the retroperitoneum through the aortic or oesophageal hiatus and sometimes through the diaphragmatic crura.^5,6,13^ Patients may present with shortness of breath, chest pain, fever, palpitations or sometimes gastroesophageal reflux due to hiatal widening.^3,5^ Occasionally, they may cause life-threatening complications due to mass effect or rupture of the pseudocysts in the pleural or pericardial cavity resulting in cardiac tamponade, circulatory failure or acute respiratory distress.^6^ Pseudocysts extending to the neck can result in dysphagia or can present as a neck swelling. In the presented case, mediastinal extension of the pseudocyst was seen through the oesophageal hiatus with further extension to the neck. The patient presented with complaints of neck stiffness and dysphagia due to mass effect on the oesophagus along with retropharyngeal and prevertebral extension.

Cross-sectional imaging plays an important role in the diagnosis of a mediastinal/retropharyngeal pseudocyst. Computed tomography demonstrates a hypodense thin-walled cystic lesion usually in the posterior mediastinum,^16^ along with its extension into the neck, when present. It is useful to evaluate the complete extent of the lesion and its relation to the visceral and vascular structures. Magnetic resonance imaging is useful to demonstrate pancreatic ductal anatomy and its communication with the pseudocyst. Ultrasonography can be used to guide the aspiration of fluid from the cyst for both diagnostic and therapeutic purposes. The presence of raised amylase levels in the fluid is highly sensitive and specific for the diagnosis of a pseudocyst of pancreatic origin.^1^

A mediastinal pseudocyst must be differentiated from a paravertebral abscess. Pseudocysts extending to the neck can be misdiagnosed as a retropharyngeal or prevertebral abscess at the preliminary examination. The clinical history, patient presentation and imaging findings aid in the diagnosis. Tuberculosis of the spine can cause paravertebral abscess formation, and patients present with long-standing history of fever, loss of appetite, weight loss, backache and localised tenderness. Typically, there is the involvement of the vertebrae adjacent to the paravertebral collections.^17^ Other differentials for mediastinal pseudocysts include neurofibroma, cystic schwannoma and other posterior mediastinal tumours, lymphoma, aortic aneurysm and extramedullary haematopoiesis.^6^ Cervical pseudocysts may mimic other cystic lesions of the neck including cystic lymph nodes, cystic lesions of the thyroid or salivary glands and lymphatic malformations.^13^

Management depends on several factors including the symptoms of the patient, location, extension of the lesion and its relation to major structures. In asymptomatic patients and patients with small lesions, medical management with octreotide may be useful. Drainage of the fluid can be performed using percutaneous or endoscopic techniques. It has been reported that the recurrence rate is lower following endoscopic drainage compared with percutaneous drainage techniques. Complications following percutaneous drainage include haemorrhage, infection and fistula formation.^18^ Surgical drainage or excision is limited to pseudocysts that fail to resolve by the above techniques or when they become complicated.^6^

### Conclusion

In any patient presenting with dysphagia along with a cystic lesion in the neck, cervical extension of a pancreatic pseudocyst must be considered as a differential under appropriate clinical settings.
